# Emergency department documentation templates: variability in template selection and association with physical examination and test ordering in dizziness presentations

**DOI:** 10.1186/1472-6963-11-65

**Published:** 2011-03-24

**Authors:** Kevin A Kerber, Timothy P Hofer, William J Meurer, A Mark Fendrick, Lewis B Morgenstern

**Affiliations:** 1 Department of Neurology, University of Michigan Health System, Ann Arbor, MI, USA; 2 Department of Internal Medicine, University of Michigan Health System, Ann Arbor, MI, USA; 3 Center of Excellence, Department of Veterans Affairs, Health Services Research & Development Service, Ann Arbor, MI, USA; 4 Department of Emergency Medicine, University of Michigan Health System, Ann Arbor, MI, USA

## Abstract

**Background:**

Clinical documentation systems, such as templates, have been associated with process utilization. The T-System emergency department (ED) templates are widely used but lacking are analyses of the templates association with processes. This system is also unique because of the many different template options available, and thus the selection of the template may also be important. We aimed to describe the selection of templates in ED dizziness presentations and to investigate the association between items on templates and process utilization.

**Methods:**

Dizziness visits were captured from a population-based study of EDs that use documentation templates. Two relevant process outcomes were assessed: head computerized tomography (CT) scan and nystagmus examination. Multivariable logistic regression was used to estimate the probability of each outcome for patients who did or did not receive a relevant-item template. Propensity scores were also used to adjust for selection effects.

**Results:**

The final cohort was 1,485 visits. Thirty-one different templates were used. Use of a template with a head CT item was associated with an increase in the adjusted probability of head CT utilization from 12.2% (95% CI, 8.9%-16.6%) to 29.3% (95% CI, 26.0%-32.9%). The adjusted probability of documentation of a nystagmus assessment increased from 12.0% (95%CI, 8.8%-16.2%) when a nystagmus-item template was not used to 95.0% (95% CI, 92.8%-96.6%) when a nystagmus-item template was used. The associations remained significant after propensity score adjustments.

**Conclusions:**

Providers use many different templates in dizziness presentations. Important differences exist in the various templates and the template that is used likely impacts process utilization, even though selection may be arbitrary. The optimal design and selection of templates may offer a feasible and effective opportunity to improve care delivery.

## Background

Clinical documentation systems have been shown to influence processes of care in many different settings[1-7]. One of the most common places that clinical documentations systems are used is the emergency department (ED). ED providers frequently use complaint-specific paper templates to document evaluation and management services. One company, T-system, Inc., makes a set of templates (e.g., "Dizziness" template, "Headache" template, "Acute Chest Pain" template) that are used by physicians to document the care encounter, and the company reports that more than 40% of EDs in the United States use these templates[8].

Although the templates are widely used, we are not aware of prior research assessing the association of the templates with processes of care. Prior research on the templates has focused on the association of the templates with time and billing outcomes[9,10]. Understanding and awareness of the effects of this particular system is important since it is widely used. The purpose of the system is primarily to aid in efficiency and billing[8]. But any item placed on a documentation form has the potential to unintentionally function as a "checklist" item. In other words, seeing the item on the template could trigger the corresponding action which might not have been performed otherwise.

Unique to the T-System templates are the numerous template options that providers have to choose from. In fact, there are more than 60 different templates (e.g., "Dizziness" template, "Headache" template, "Acute Chest Pain" template) that are used by physicians to document the care encounter. To our knowledge, prior research has not reported on the variability in use of individual templates within a population of a symptom presentation like dizziness. Though there is a "Dizziness" template, there are also other templates that could reasonably be selected in primary dizziness presentations (e.g., "Nausea, Vomiting, Diarrhea" template, "General Adult" template, "Neuro Deficit" template) and other templates that could be selected if dizziness was one of several symptoms, which is a common scenario[11]. If there is wide variation in template selection and also important differences in the templates, then future refinements of the system would need to consider selection of the template as an important factor in optimizing its effects.

In a population-based study of ED dizziness visits, we discovered that all participating EDs use the T-System complaint-specific templates as the principal form of physician documentation. Because of considerable heterogeneity in the type of template used within this group and important differences in the items on different templates, we had an opportunity to describe variability in the selection of templates and also to test a hypothesis that the template type used to document care is associated with the documentation of processes of care. We selected two processes of care to study the potential association: head computerized tomography (CT) scan utilization and nystagmus assessment documentation. These processes were chosen because they are both important topics in dizziness evaluations and because items for these processes only appear on a subset of the templates. Head CT scan use is an important topic because it is widely regarded as overused in the assessment of dizziness patients[11,12]. Nystagmus assessment is an important topic because nystagmus is the hallmark indicator of vestibular dysfunction and thus can be the crucial element in making a diagnosis[13,14]. Dizziness presentations are very common and heterogeneous, and important differences are likely to exist between dizziness patients that receive different complaint-specific templates. To control for this important source of selection effects, propensity score matching was used[15]. If templates do impact care delivered, then the optimal design and selection of templates may be a feasible way of optimizing care.

## Methods

### Study setting and Data Collection

The Dizziness Evaluation and Treatment in Corpus Christi, Texas (DETECT) Project is a population-based, emergency department, dizziness surveillance study in Nueces County, Texas. The ultimate aim of the study is to define the impact of dizziness in terms of numbers and healthcare utilization, and to search for opportunities to optimize care. The county is served by six adult care EDs. The study was approved by the relevant institutional review boards (i.e., The University of Michigan and the participating EDs in Corpus Christi) and granted a HIPPA waiver of informed consent. Prospective active case ascertainment is utilized to review recent ED presentations. Dizziness visits are identified by a trained abstractor who screens ED logs for any of the following reason for visit terms: dizziness, imbalance, or vertigo. The abstractor underwent training procedures and certification in the collection of data and in data entry, and was blinded to the current study question. On-going quality assurance mechanisms for data collection are in place. For the purposes of the current study, patient visits were identified from January 15, 2008 through January 14, 2009.

### Inclusion criteria

The study population included all visits for dizziness to one of the Nueces County EDs. Exclusion criteria were age <18 years, principal residency outside of Nueces County, institutionalized individuals, trauma presentations, and patients leaving before being seen by a health care provider.

### ED template type exposure and Outcomes

All EDs participating in this study use complaint-specific templates produced by T-System, Inc.,[8] as the primary record of the physician encounter. The T-System templates are a set of 60 different chief complaint-specific paper templates. Most templates are two pages in length and each template was developed to address presentation components relevant to the specific chief complaint. Utilizing the pre-printed items on the template, characteristics of the patient presentation and assessments rendered (i.e., evaluation and management) are documented by ED providers during the course of care using circles, checks, and backslashes. Each section of the template also has blank space where physicians can hand write additional information. For the purposes of this study we coded each of the complaint-specific template types used in this study as a head CT-item template and/or a nystagmus-item template, if the template type contained relevant items pre-printed on the form (Table 1). We excluded visits when a template was not used to document the encounter (i.e., the encounter was instead documented with a handwritten or dictated note) or when the template was of poor quality such that information could not be abstracted.

**Table 1:** Coding scheme for template types.

	Head CT-item template	Nystagmus-item template
Relevant item(s)*	"Head CT" or "CT Scan head"	"Nystagmus"
Template types containing the relevant item(s)	Altered Mental Status; Dizziness; Fall; Headache; Neurological Deficit; Seizure; and Syncope and Near-Syncope	Dizziness; Psych Disorder, Suicide, Overdose

CT = computerized tomography

* Relevant items = items that were pre-printed onto the complaint-specific templates.

The primary outcomes considered in this study were documentation of head computerized tomography (CT) and nystagmus assessment. A head CT was determined to have been performed if a head CT result was recorded on the template or a head CT report accompanied the ED record. Visits were considered as having documentation of a nystagmus assessment if the completed template indicated the assessment was performed. To count as performed, the template needed to either have the relevant item checked/circled/slashed (if it was a pre-printed item) or have the item and results of the assessment handwritten on to the template. When these criteria were not met, the process was counted as not performed.

### Covariates

Covariates were included that were thought to be associated with the use of template types, as well as variables thought to be associated with the utilization of the processes of interest. These included sociodemographic variables (age, gender, race-ethnicity, insurance status), hospital, type of dizziness symptom, dizziness presentation type, number of medical symptoms (inclusive of dizziness symptoms, pain, palpitations, fatigue, generalized weakness, nausea, vomiting, shortness of breath), number of other neurologic signs or symptoms, number of stroke risk factors, and admission status. A clinical item (i.e., symptom, exam sign, stroke risk factor) was considered to be absent if there was no mention of it being present. The type of dizziness symptom was categorized using a hierarchy established a priori whereby mention of vertigo in the template was selected over other dizziness symptoms, and subsequently the hierarchy followed as such: imbalance, non-vestibular types of dizziness (i.e., lightheadedness, fainting, or psychological dizziness), and dizziness not otherwise specified. The dizziness presentation type was also categorized using a common categorization scheme because of the differences in potential etiologies relative to the presentation characteristics[13]. The presentation categories were determined by the abstractor who searched the template for key descriptors about the characteristics of the presentation and classified each case using predefined criteria. Five categories of dizziness presentations were used: acute (i.e., ≤ 7 days from onset) constant dizziness, recurrent spontaneous attacks of dizziness, recurrent positionally triggered attacks of dizziness, subacute or chronic (>7 days from onset) constant dizziness, and dizziness as an accompaniment symptom. Presentations were categorized as "dizziness as an accompaniment" when dizziness was not the principal symptom. As a data reduction method for the project, visits categorized with dizziness as an accompaniment presentation were no longer fully abstracted beginning on September 12, 2008. Thus visits categorized with dizziness as an accompaniment on or after that date were not included in the current analysis.

### Statistical analysis

Logistic regression models were used to compare the odds of documentation of each process among patient visits having a template that contained the relevant pre-printed items compared to patient visits that did not have a template with relevant pre-printed items, controlling for covariates. Interaction terms of template type and each other covariate were tested. In the head CT models, a strong interaction of the template type with the dizziness presentation type was found so the interaction terms were retained in the final model. The c-statistic of each model demonstrated good to excellent discrimination (nystagmus model c-statistic, 0.9704; head CT model c-statistic, 0.8093) and Hosmer-Lemeshow tables showed excellent calibration for each model. The logistic regression models were used to generate the predicted probability of having the relevant process documented or performed by template type while holding all other covariates at their means.

Repeated visits were approached by limiting the population to only the first visit. A secondary analysis was performed including all visits and controlling for repeated visit effects (multivariable generalized estimating equation model) but results did not differ.

Propensity score analysis was performed to further assess the comparability of the treatment groups. Standard methods for propensity score development were used and a detailed description of these methods is provided in Additional file 1. All analyses were performed using STATA 10.0 (StataCorp, College Station, Texas).

## Results

Out of 1,593 visits for dizziness, there were 1,488 unique patients. After excluding three visits that had a dictated or hand-written note rather than a template, the final cohort was comprised of 1,485 visits. No visits were excluded because of inability to abstract information from the template. The median age of the cohort was 49.7 years (range, 18-96 years), and 974 (65.6%) were female. Table 2 shows the characteristics of the population.

**Table 2:** Baseline characteristics of the Study Population

	Population receiving a template with a head CT item (n = 1,036)	Population receiving a template without a head CT item (n = 449)	Population receiving a template with a nystagmus item (n = 872)	Population receiving a template without nystagmus item (n = 613)
Age, mean ± SD, y	53.9 ± 19.4	44.8 ± 18.4	54.5 ± 19.0	46.4 ± 19.4
Female (%)	64.9%	67.3%	65.1%	66.2%
Race-ethnicity (%)				
Non-Hispanic White	26.2%	27.2%	26.7%	26.1%
Mexican American	67.3%	67.9%	67.3%	67.7%
Other or unknown	6.6%	4.9%	6.0%	6.2%
Insurance, any (%)	79.0%	69.5%	80.0%	70.6%
Dizziness Presentation				
Accompaniment	14.0%	73.1%	5.4%	69.5%
Acute Severe	45.0%	16.3%	48.4%	19.1%
Recurrent Positional	7.6%	2.2%	8.6%	2.3%
Recurrent Spontaneous	27.0%	6.0%	30.5%	6.7%
Subacute to Chronic	6.4%	2.5%	7.1%	2.5%
Dizziness Symptom				
Lightheaded or other	23.1%	12.7%	24.1%	14.0%
Dizziness NOS	20.2%	79.5%	13.3%	73.4%
Imbalance	15.4%	1.3%	15.7%	4.7%
Vertigo	41.3%	6.5%	46.9%	7.8%
Number of medical symptoms				
0 - 1	5.9%	13.6%	4.9%	13.0%
2	17.6%	24.9%	17.3%	23.3%
3	23.4%	25.8%	22.6%	26.3%
4	20.5%	19.6%	21.8%	17.9%
≥5	32.7%	16.0%	33.4%	19.6%
Number of neurological symptoms or signs				
0	66.3%	82.6%	73.5%	68.2%
1	26.9%	14.5%	22.0%	24.8%
≥2	6.7%	2.9%	4.5%	7.0%
Number of stroke risk factors				
0	30.8%	37.2%	29.5%	37.4%
1	31.9%	33.4%	32.9%	31.5%
2	19.7%	18.7%	19.6%	18.8%
≥3	17.7%	10.7%	17.8%	12.4%

Abbreviations: CT, Computerized tomography; NOS, not otherwise specified.

A total of 31 different templates were used in this population. The most common template used was the 'Dizziness' template (53% visits), followed by 'Nausea, Vomiting, and Diarrhea' template (6.5%), 'General Adult' template (5.7%), 'Headache' template (5.3%), and 'Abdominal Pain' template (4.1%). A head CT was performed in 28.5% of visits, and a nystagmus assessment was documented in 59.8% of visits.

**Figure 1:**
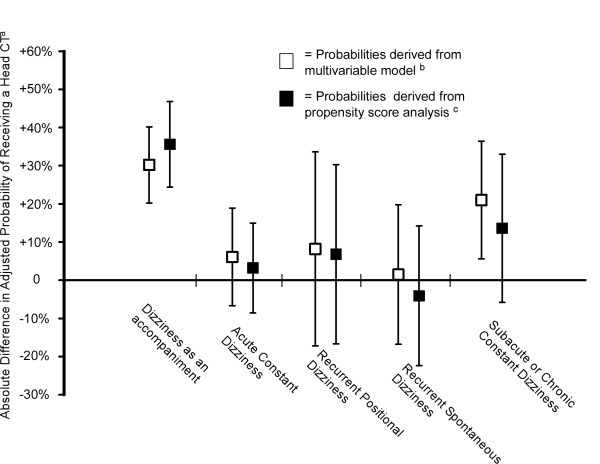
**Absolute difference in adjusted probability of receiving a head computerized tomography (CT) scan when a head CT-item template was used compared to when a head CT-item template was not used. Error bars indicate 95% confidence intervals**. ^a ^Absolute difference calculated as follows: Adjusted probability of receiving a Head CT when a template with a pre-printed head CT item is used MINUS adjusted probability of receiving a head CT when a template with a pre-printed head CT item is not used. ^b ^Probability of head CT was derived from a logistic regression model with head CT performance as the dependent variable and the following independent variables: socio-demographic variables (age, gender, race-ethnicity, insurance status), hospital, type of dizziness symptom, dizziness presentation type, number of medical symptoms, number of other neurological signs or symptoms, number of stroke risk factors, admission status, head CT-item template, and the interaction terms of head CT-item template with dizziness presentation type. Probabilities were calculated with all other variables in the model held constant at the population means. ^c ^Probability of head CT was derived from a logistic regression model with the dependent variable of head CT performance and the following independent variables: head CT-item template, dizziness presentation type, the interaction terms of head CT-item template with dizziness presentation type, and the propensity score quintile. The propensity scores were derived from a logistic regression model with head CT-item template as the dependent variable and the same independent variables as in the first model.

A head CT was performed in 38.8% (402/1036) of the visits that received a template with a head CT item, compared with only 11.1% (50/449) of those not receiving a template with a head CT item. Even after adjusting for the covariates in the multivariable model, the difference remained substantial with the probability of head CT performance of 29.3% (95% CI, 26.0%-32.9%) when a template with a head CT item is used compared to only 12.2% (95% CI, 8.9%-16.6%) when template without a head CT item is used. Testing for interaction effects found a significant interaction of the template type with the dizziness presentation type, such that the overall association of the template type with head CT performance was mostly driven by the "dizziness as an accompaniment" presentation type. In the dizziness as an accompaniment presentation group the probability of receiving a head CT was 38.5% (95% CI, 29.7%-48.2%) if a template with a head CT item was used compared with 8.4% (95% CI, 5.2%-13.3%) if a template without a head CT item was used. The other presentation categories demonstrated trends favoring an association of head CT performance with head CT template use (Table 3), and a separate analysis conducted after excluding the "dizziness as an accompaniment" patients (i.e., population of all dizziness presentations except those with dizziness as an accompaniment) found that the probability of receiving a head CT was 31.8% (95% CI, 28.5%-35.4%) if the patient received a template with a head CT item versus 23.4% (95% CI, 15.7%-33.3%) if the patient did not, with all other covariates held at their means. Propensity score adjustments led to slight changes in the effects but no substantial changes in the overall inferences (Table 3). The absolute differences in the adjusted probability of receiving the head CT are shown in Figure 1.

**Table 3:** Probability of receiving a head CT based on head CT-item template, adjusted by multivariable analysis and propensity score.

	MV model adjustment		Propensity score adjustment	
	Received head CT-item template	Did not receive head CT-item template	Received head CT-item template	Did not receive head CT-item template
Dizziness Presentation Type				
Accompaniment	38.5% (29.7%-48.2%)	8.4% (5.2%-13.3%)	48.8% (39.2%-58.5%)	13.2% (7.6%-21.8%)
Acute Constant	33.9% (29.0%-39.1%)	27.8% (17.7%-40.8%)	31.3% (26.1%-37.1%)	28.1% (18.6%-40.1%)
Recurrent positional	25.2% (16.4%-36.7%)	17.0% (3.8%-51.7%)	23.5% (15.6%-33.8%)	16.7% (3.9%-49.3%)
Recurrent spontaneous	25.8% (20.4%-32.1%)	24.4% (11.2%-45.0%)	24.8% (19.4%-31.1%)	29.0% (14.8%-48.9%)
Subacute to chronic constant	25.7% (15.8%-38.9%)	4.7% (0.5%-31.4%)	22.6% (14.3%-33.7%)	8.9% (1.2%-43.8%)

Abbreviations: CT, computerized tomography; MV, multivariable logistic regression.

**Figure 2:**
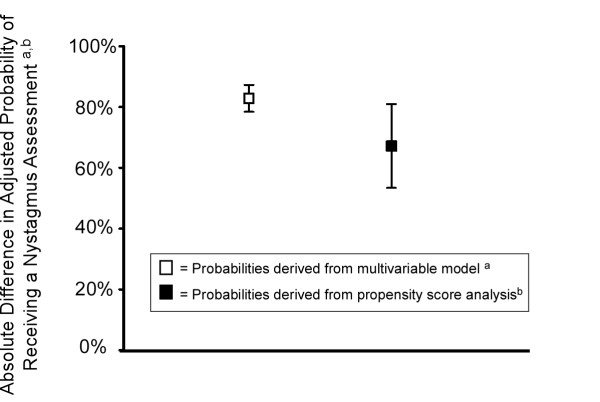
**Absolute difference in adjusted probability of documentation of a nystagmus assessment when a template with a nystagmus item was used compared to when a template with a nystagmus item was not used. Error bars represent 95% confidence intervals**. ^a ^Absolute difference for the multivariable model was calculated as follows: Adjusted probability of receiving a nystagmus assessment when a template with a nystagmus item is used MINUS adjusted probability of receiving a nystagmus assessment when a template with a nystagmus item is not used. Probabilities of nystagmus assessment was derived from a logistic regression model with nystagmus assessment as the dependent variable and the following independent variables: socio-demographic variables (age, gender, race-ethnicity, insurance status), hospital, type of dizziness symptom, dizziness presentation type, number of medical symptoms, number of other neurologic signs or symptoms, number of stroke risk factors, admission status, and nystagmus item template. Probabilities were calculated with all other variables in the model held constant at the population means. ^b ^Absolute difference for the propensity score analysis was calculated using nearest neighbor propensity score matching. First a propensity score was derived (see Additional file 1). Then, the propensity score was used for nearest neighbor matching to calculate the adjusted absolute difference in the probability of receiving the nystagmus assessment when a nystagmus item template was used compared to when a nystagmus item template was not used. Covariates used in the model were the following: socio-demographic variables (age, gender, race-ethnicity, insurance status), hospital, type of dizziness symptom, dizziness presentation type, number of medical symptoms, number of other neurologic signs or symptoms, number of stroke risk factors, admission status, and nystagmus item template.

A nystagmus assessment was documented in 95.6% (834/872) of visits having a nystagmus item template and only 8.8% (54/613) of visits without a nystagmus item template. Even after adjusting for all covariates in the model (all covariates set to their mean), the probability of documentation of nystagmus remained high (95.0%, 95% CI, 92.8%-96.6%) when a nystagmus item template was used and low (12.0%, 95%CI, 8.8%-16.2%) when the template used did not contain a nystagmus item (Figure 2). After matching visits by propensity score, the visits having a nystagmus template remained substantially associated with documentation of a nystagmus assessment. The absolute difference in the probability of receiving a nystagmus assessment calculated by the propensity score analysis was 67.8% (95% CI, 54.6%-81.1%) when a nystagmus-item template was used compared to when a nystagmus-item template was not used.

## Discussion

The optimal design and selection of templates may offer a feasible and effective opportunity to improve efficiency and reduce variability in healthcare. The finding that systems of clinical documentation are associated with clinical care is not new[1-7]. Across a wide spectrum of clinical topics and study designs, these associations have been reported. The current study contributes to the evidence base on the relationship of documentation systems to clinical care for a couple of reasons. First, the template system studied, T-System, Inc., is already widely used (40% of EDs)[8]. Thus, it is important to explore any unintended effects of the system since the real world impact could be substantial. Another unique feature of this study is that the documentation system has a large number of template options. For each patient, a provider must choose from more than 60 different chief-complaint focused templates. As a result, we had the opportunity to study variability in template choice within a large population of similar symptom presentations.

Template systems could be an important factor in the efforts to optimize CT scan use because we found that a head CT template item is associated with head CT scan utilization in this population of dizziness presentations. Though the association was concentrated in the presentation of "dizziness as an accompaniment" group, the trends found in other groups may also be relevant considering that there are more than 2.5 million annual ED dizziness presentations in the United States [11] and 40% of the EDs use these templates[8]. Finding the association of template item with CT use is particularly provocative and timely because a strong sentiment exists that CT studies are overused in dizziness presentations[12,16-18]. Head CT scans may be particularly overused in dizziness presentations because nearly 30% of dizziness visits in the ED receive one even though serious central nervous system causes are uncommon,[11] the sensitivity of CT is dismal for the most common central cause (ischemic stroke),[19] and the test is associated with important harms including cost, radiation exposure, and time in the ED[12,20,21]. We don't know what the optimal rate of head CT use should be for dizziness presentations, but even a small reduction in the nearly 30% use rate could have an important impact on reducing the net "harms" of the test with very little or no trade off in lost net benefit. Changes in template design should be feasible because these changes should be spared from the barriers to change that impact the many other non-clinical factors related to the use of imaging studies[16,20,22].

The optimal design and use of templates might also help to optimize the bedside assessment of the patient with dizziness. Nystagmus assessment documentation had a strong association with whether the nystagmus item was on the template or not. Nystagmus assessments are an important aspect of the clinical evaluation of dizziness patients because the findings can be the critical component in discriminating among causes of dizziness[13,14]. Prior research demonstrates the shortcomings of differentiating causes of dizziness by the patient's description of the symptom and even by extensive diagnostic testing[23,24]. Nystagmus, however, is the hallmark sign of a vestibular disorder[13,14]. The presence or absence of nystagmus and its pattern are key to determining the most likely etiology or localization in dizziness presentations. Some patterns of nystagmus (e.g., uni-directional horizontal nystagmus) are highly indicative of a benign peripheral vestibular disorder in the appropriate clinical context, whereas other patterns (e.g., bi-directional or pure vertical nystagmus) are highly indicative of a central disorder[14]. No nystagmus in an acute dizziness presentation would make any vestibular involvement unlikely. As a result the nystagmus assessment plays an important role in the diagnosis and resulting management decisions. Because the current results suggest a strong association of template-item type with which patients have a nystagmus assessment, optimal template examination items may be a way to reduce variability of important examination assessments. An important limitation to the findings of the examination assessments is that we cannot confirm that documented exam components were actually performed.

The associations between template items and processes suggest that template items might function like a "checklist" item even though this is not the intention of the item placement. The primary intention of the template system is to improve time, efficiency, and billing accuracy[8]. But an item placed on a template could have the unintended effect of functioning like a checklist item by reminding the provider to consider the process[25]. In some cases this may be an unintended positive effect, whereas in other cases it may be an unintended negative effect.

We found that many different template types were used in this population. The large number of template types used suggests that the template selection may be more arbitrary than systematic. Though the "Dizziness" template was by far the most commonly used, more than 30 different template types were used throughout the whole population. The propensity scores were well balanced on many different important variables (about 95% of the visits were in the area of common support in the propensity score analysis) indicating substantial similarities in the characteristics of visits receiving different template types. We do not know why so many different templates were used. Some possible factors include the non-specific nature of the "dizziness" label and the fact that dizziness frequently co-occurs with other symptoms[11]. When important differences exist in the content of the various templates, then the selection of the template by the physician may be just as important as the design of the template by the company. This in turn leads to the important question: What happens if the wrong template is picked? The results of this study suggest that the patient would be more likely to receive processes unique to the "wrong" template and less likely to receive processes unique to the "right" template. These differences could influence the efficiency and effectiveness of care.

What contributes to the template selection is not clear. Further research is necessary to understand this factor. In some cases, the template may be selected by the triage staff or based on triage staff documentation. Some physicians may have a preference for certain templates and select the template based on its content relative to what they already plan to do.

We did not find an "ideal" template for dizziness presentations in this population, and thus it would be difficult to judge the appropriateness of the template selection. For example, the "Dizziness" template was only one of two templates that had nystagmus as a pre-printed item but this template also had a head CT item. Thus a dizziness presentation receiving the "Dizziness" template was more likely to receive a nystagmus assessment (unintended benefit), but also was more likely to receive a head CT (unintended consequence). All of the template options that did not have a head CT item also did not have a nystagmus assessment item. Ultimately, these issues have a large potential to be resolved by using an electronic template that can adjust upcoming items (e.g., exam components, management options) based on details entered about the clinical presentation, thus reducing the effect of selection of a paper template.

This study was performed in a representative community and the results may not be generalizable to other settings. The analysis was not adjusted for physician level variability because physician identifiers were not collected. Physician level information should be considered in future studies of this type. This study was limited as it was based on medical record review. It remains possible that clinical information was obtained by treating physicians but was not documented. Nevertheless, prior research has demonstrated acceptable concordance between documentation in the medical record and actual performance, as assessed by direction observation or videotapes[26,27]. Because there are no consensus guidelines on the use of head CT in dizziness presentations, the appropriateness of use of the test could not be assessed. No randomized controlled trials have proven the benefit of a nystagmus assessment on patient outcomes, though the same could be said for most individual bedside assessment components. Ultimately, the cause and effect of template-items on care delivered can only be determined by randomized controlled trials which would be an important future step in efforts to optimize care. Future studies would also be important to assess whether unintended template effects contribute to either outcome benefits or outcome consequences.

## Conclusion

This study found that many different complaint-specific templates are used by providers to document care of ED dizziness presentations. In addition, we found an association of template type used to document care with the processes of care delivered. The template type was associated with head CT utilization. Documentation of a nystagmus examination was strongly associated with whether a nystagmus item was included as a pre-printed item on the template used to document care. Optimal template item design and selection is likely to be an important area of future research since items impact care and selection of templates may be arbitrary. The more widespread adoption of electronic medical records will increase the use of template-like documentation systems enabling a potentially large opportunity to tie documentation and ordering of tests to efficient and effective algorithms of care. An electronic template also has the potential to reduce selection effects. The optimal design and selection of templates may offer a feasible and effective opportunity to improve care delivery.

## Competing interests

The authors declare that they have no competing interests.

## Authors' contributions

KAK, LBM, TPH, AMF, and WJM made substantial contributions to the study concept and design. KAK and LBM collected the data. KAK and TPH performed the statistical analysis. KAK, TPH, AMF, and LBM made substantial contributions to the interpretation of data. KAK wrote the manuscript. KAK, LBM, TPH, AMF, and WJM made substantial contributions to the critical revisions of the manuscript. All authors read and approved the final manuscript.

## Pre-publication history

The pre-publication history for this paper can be accessed here:

http://www.biomedcentral.com/1472-6963/11/65/prepub

## Supplementary Material

Additional file 1 **Propensity score analysis methods**. This additional file describes the methods used for the propensity score analysis Click here for file
