# Supplementary material for: Emergency department documentation templates: variability in template selection and association with physical examination and test ordering in dizziness presentations
Source: BMC Health Serv Res. 2011 Mar 24;11:65. doi: 10.1186/1472-6963-11-65 (PMC3073892; doi:10.1186/1472-6963-11-65)
Supplement: Additional file 1 — Propensity score analysis methods. This additional file describes the methods used for the propensity score analysis [file 1472-6963-11-65-S1.DOC]

*Propensity score analysis methods:*

Propensity score analysis was performed to further assess the comparability of the treatment groups. Multivariable logistic regression was used to model the type of template type selected (dependent variable) as a function of the variables available to the ED physician when selecting the template and that were likely to influence the template type used in our cohort. The same sets of covariates described in the multivariable model analysis were available and all were included in the regression as there is no disadvantage to over-fitting in the propensity model.^1^ The full regression model was then used to generate the predicted probability of receiving the template of interest for each visit (i.e., the “propensity score”), which ranges between 0 and 1. We followed the recommended method of assessing the propensity score model and limiting the cohort to the area of common support.^2^ The propensity score model is assessed by whether it creates balance in the covariates between the treatment and control groups. The current analysis achieved balance with six quantiles, with all six quantiles balanced on mean propensity scores and means of each variable. The common support cohort was 1,446 (2.6% of visits dropped) for the head CT analysis and 1,408 (5.2% of visits dropped) for the nystagmus analysis.

The adjusted difference in the predicted probability of receiving the outcome (in this case, documentation of process of interest) was then calculated by comparing visits that received the relevant templates of interest to visits that did not. Because the head CT analysis demonstrated that the effect of the template type on head CT process was modified by the dizziness presentation type, the absolute difference in the predicted probability of head CT assessment based on template type was estimated for each presentation type from a logistic regression model with head CT assessment as the dependent variable and the following independent variables: head CT item template, dizziness presentation type, interaction terms of head CT item template with dizziness presentation type, and propensity score quintile. Nearest neighbor propensity score matching was used for the nystagmus analyses. This method matches each treated visit to the untreated visit having the closest propensity score and was applied with replacement.^2^ Bootstrapping was used to calculate the 95% confidence intervals of the effects.

1. D'Agostino RB, Jr. Propensity score methods for bias reduction in the comparison of a treatment to a non-randomized control group. Stat Med 1998;17:2265-81.

2. Becker SO, Ichino A. Estimation of average treatment effects based on propensity scores. The Stata Journal 2002;2:358-77.
